# Frailty score and outcomes of patients undergoing vascular surgery and amputation: A systematic review and meta-analysis

**DOI:** 10.3389/fcvm.2023.1065779

**Published:** 2023-01-25

**Authors:** Shujie Chen, Riley Dunn, Mark Jackson, Nicola Morley, Jing Sun

**Affiliations:** ^1^School of Medicine and Dentistry, Griffith University, Gold Coast, QLD, Australia; ^2^School of Pharmacy and Medical Sciences, Griffith University, Nathan, QLD, Australia; ^3^Department of Vascular Surgery, Gold Coast University Hospital, Gold Coast, QLD, Australia; ^4^Institute for Integrated and Intelligent Systems, Griffith University, Gold Coast, QLD, Australia

**Keywords:** frailty, frailty scores, vascular surgery, vascular disease, amputations, health outcomes

## Abstract

**Introduction:**

Frailty is associated with adverse postoperative health outcomes, including increased mortality, longer length of stay, higher rehospitalization, and other complications. There are many frailty assessment tools are to assess the level of frailty in vascular surgery patients. The aim of this study was to perform a systematic review and meta-analysis to assess the association between the frailty levels described by different frailty scores and adverse postoperative health outcomes among hospitalized vascular surgery patients and patients undergoing amputation.

**Methods:**

Studies utilizing frailty scores and similar frailty assessment tools to describe frailty and investigate the association between frailty and health outcomes were searched. The primary outcomes of this study were in-hospital mortality, postdischarge mortality, length of hospital stay, rehospitalization, and discharge location. Additional outcomes included postoperative myocardial infarction, postoperative renal failure, cerebrovascular accident and stroke, comorbidities, and estimated glomerular filtration rate (eGFR) levels. Joanna Briggs Institute (JBI) Critical Appraisal Tools were used for quality assessment.

**Results:**

In total, 24 studies with 1,886,611 participants were included in the final analysis. The overall results found that higher in-hospital mortality and postdischarge mortality were significantly associated with frailty. Frailty was also found to be significantly associated with a longer length of hospital stay, higher rehospitalization, and higher likelihood of non-home discharge. In addition, the results also showed that frailty was significantly associated with all kinds of comorbidities investigated, except chronic kidney disease. However, lower eGFR levels were significantly associated with frailty.

**Conclusion:**

Among patients who underwent all types of vascular surgery and those who underwent amputations, assessment of frailty was significantly associated with adverse postoperative outcomes and multiple comorbidities.

**Systematic review registration:**

https://www.crd.york.ac.uk/PROSPERO/display_record.php?RecordID=336374, identifier CRD42022336374.

## Introduction

All-cause mortality is increasingly higher in both male and female populations with peripheral vascular disease (PVD) (1). Males with peripheral arterial disease also have poorer performance in subjective measures of lower extremity functions (2). Among patients with vascular diseases, those with critical limb ischemia (CLI) have a preoperative functional status closely associated with decreased short-term survival and an increased number of comorbidities (3). The development of CLI can be associated with both diabetic foot (4) and other types of vascular disease (5), which are both linked to increased comorbidities and decreased survival rates compared to the healthy population with the same age profile (6). Traditionally, frailty is usually considered to be associated with aging and can lead to adverse outcomes, such as disability, vulnerability, and death (7).

Frailty is a characteristic described as the increasing vulnerability of a patient to stressors and the declining function in multiple physiological systems (8–10). It is thought to be associated with increased mortality, falls, and longer length of stay (LOS) for hospitalized patients (8–10). The distinct features of frailty also include a decrease in physiological reserve, impairment in multiple physiological systems, and reduced function and ability to retrieve physiological homeostasis after experiencing stressors and disturbances such as disease, operations, chronic conditions, and accidents (11). These features make frailty easy to distinguish from similar conditions such as disability, chronic diseases, and other comorbidities. For example, “disability” refers to limitations and dependence on mobility and life skills. However, an individual defined as “disabled” can have normally functioning physiological systems and no reduction in physiological reserve thus not being frail (10). This difference is adaptable even among older populations in which frailty occurs more often. An individual with “comorbidities” is someone with multiple chronic conditions such as diabetes, hypertension, abnormal blood cholesterol level, chronic kidney conditions, chronic obstructive pulmonary disease (COPD), cardiovascular disease (CVD), and stroke. However, the presence of multiple chronic conditions alone is not enough to identify such an individual as frail, which is associated more with impairment in physiological reserve and physiological systems (10). All these features make frailty a distinct status to describe the clinical conditions of an individual, and the condition has been shown associated with multiple adverse postoperative health outcomes (12).

Fried et al. (8) has proposed a frailty phenotype model that describes frailty as meeting three or more of the five phenotypic criteria: weakness as measured by low grip strength, slowness measured by slowed walking speed, a low level of physical activity, low energy or self-reported exhaustion, and unintentional weight loss. This model, known as “Fried Criteria,” are commonly used in the geriatric population, and forms the base of the development of multiple frailty measurement tools. Frail patients, are less likely to recover well from acute stressors and are thus more prone to adverse health outcomes after major surgical procedures (13). Among different surgical disciplines, vascular surgery patients are typically older and have significantly increased comorbidities (14). These characteristics make them likelier to experience adverse postoperative conditions (13, 15).

While there is growing recognition that frailty score are associated with the outcome of hospitalized patients undergoing vascular surgeries and amputations (16, 17), few studies have been conducted that summarize and analyze the findings of both vascular surgery patients and amputation patients. Considering the demographic similarities and overlaps between the two patient profiles, we included both patient populations in our review.

The aim of this study was to systematically review and analyze the associations between frailty levels described by different frailty scores and adverse postoperative health outcomes among hospitalized vascular surgery patients and patients who underwent amputations only.

## Materials and methods

The systematic review and meta-analysis were performed strictly following the Reporting Items for Systematic Review and Meta-Analysis (PRISMA) approach (18). The protocol for this study was registered with the International Prospective Register of Systematic Reviews (CRD42022336374).

### Search strategy

The literature search was independently performed by two researchers (SC and RD). Six databases were included in the original search: Medline, PubMed, Embase, Cochrane Library, Scopus, and PsycINFO. The following keywords were used to search relevant articles: [(“frailty” OR “frailty score”) AND (“vascular surgery” OR “major vascular surgery”) AND (“amputation” OR “diabetes” OR “diabetic”) AND (“mortality”)]. Reference lists from the systematic review and meta-analysis published for the relevant topic were also reviewed to include as many relevant articles as possible. Additional articles from other sources, including Google Scholar and the Griffith University library, were also included.

### Inclusion and exclusion criteria

As frailty is more common in the elderly population with some reports in younger ages, we have put our focus on the adult population who have been hospitalized for vascular surgeries. As we want to see how frailty would have an impact on perioperative health outcomes, the exposure and comparator in our criteria were whether the patient is frail or not, and any perioperative related health outcomes including chronic conditions were our outcomes of interest. Considering vascular surgery covers a very broad range of surgeries with each having a different impact on patients, a study design that measured each surgery separately is very hard to achieve due to ethical and practical reasons. In addition, we would like to see if frailty would have a significant association with health outcomes in vascular patients regardless of the actual surgery type, we have considered all open and endovascular surgeries vascular disease patients would possibly take to treat their conditions relevant in our meta analysis. These surgeries include elective open abdominal aortic aneurysm repair, endovascular aneurysm repair, thoracic endovascular aortic repair, suprainguinal bypass, infrainguinal bypass, carotid endarterectomy, peripheral vascular intervention, transcatheter mitral valve repair, carotid endarterectomy, lower extremity revascularization, and major lower extremity amputation and/or all kinds of amputations. More observational study designs including case control studies and cohort studies are expected due to ethical difficulties in conducting randomized control trials in this area, but randomized control trials were also considered in this analysis if any have shown up during the study selection process.

The inclusion criteria for this study were as follows:

1. Hospitalized patients included both male and female patients aged 18 years and older.
2. Participants had undergone vascular surgeries defined previously.
3. The exposure measures were frailty using the Clinical Frailty Scale (CFS) or other frailty assessment tools in perioperative assessments. Comparison groups in the study were the “frail group” and “non-frail group.”
4. The primary outcomes of the study were in-hospital and postdischarge mortality, LOS, rehospitalization, and discharge location. Secondary outcomes were HbA1c level, glomerular filtration rate (GFR) level, postoperative myocardial infarction, cognitive impairment, smoking history, and comorbidities, including stroke, renal failure, hypertension, cardiovascular disease (CVD), PVD, diabetes, chronic obstructive pulmonary disease (COPD), and chronic kidney disease (CKD).
5. The study design was randomized control trials, cohort studies, or case control studies.

The exclusion criteria for this study were as follows:

1. Frailty was not measured in the perioperative period.
2. The study included self reported cases and community patients.
3. Participants were not grouped based on frailty level.
4. The results were only reported in graphs.
5. There were insufficient data reported for analysis.

Frailty scores were assessed using different assessment tools, but the exposure of interest in this study was frailty status. Because the grouping gave us information on how frailty level was associated with the health outcomes of patients who underwent vascular surgeries or amputations, studies that did not group the participants based on their frailty status (e.g., frail or non-frail) were excluded. Multiple frailty assessment tools were used to assess the level of frailty in both inpatient clinical settings and outpatient settings (13–15). Among these tools, many have numerated frailty to a frailty score that shows individuals’ frailty levels (13–15). Some of the commonly used frailty scores include the CFS (19), the Modified Frailty Index (mFI) (20, 21), the FRAIL scale (22), and the Rockwood Frailty Index (23). A typical frailty score considers multiple domains that include dimensions in physical fitness, psychological outcomes, and social abilities (13, 14). These scores evaluate the overall vulnerability and fitness of the patient to provide data that represent the level of frailty. Typically, the higher the frailty score, the frailer the patient. A cutoff score is applied to most of the clinically used frailty scores, with patients scoring less than the cutoff score being non frail and patients scoring higher than the cutoff score being frail (13–15, 19–21, 23–25). Some frailty scores, such as the Hospital Frailty Risk Score (HFRS), further identify frail patients with intermediate frailty and high frailty (26). Different frailty scores have their own frailty cutoff scores and definitions. For instance, the CFS is a nine point assessment scale for which each point has its own definition. Typically, a CFS score of 5 or more is deemed frail (19). The Rockwood Frailty Index is calculated based on the number of deficits that a patient has out of a list of potential deficits. Patients scoring less than 0.1 are classified as robust, patients scoring 0.1–0.2 are prefrail, patients scoring 0.2 to less than 0.25 are approaching frailty, and patients scoring 0.25 and higher are frail (23).

### Quality assessment

Quality assessment was independently conducted by the two reviewers using the JBI Critical Appraisal Tool Checklist for Case Control Studies and the Checklist for Cohort Studies (27). The full checklist can be found at the following link: https://jbi.global/critical-appraisal-tools. The Checklist for Case Control Studies includes 10 domains: comparable grouping, appropriately matching cases and controls, the same identification criteria for cases and controls, valid measurement of exposure, the same way of measuring exposure between cases and controls, identified confounding factors, strategies to deal with confounding factors, valid measurement of outcomes, sufficient time of followup, and appropriate statistical analysis. The Checklist for Cohort Studies contains 11 domains: comparable grouping, similar measurement of exposure to assign people to both exposed and unexposed groups, valid and reliable measurement of exposure, identified confounding factors, stated strategies to deal with confounding factors, groups and/or participants free of outcomes at the starts of the study, valid and reliable measurement of outcomes, sufficient reported follow up time, completed follow up or described and explored loss of follow up, valid strategies to address incomplete follow up, and appropriate statistical analysis. A cutoff score of 5 was applied to include both medium quality and high quality studies.

### Data extraction

Data extraction was completed by the primary reviewer (SC), with a second reviewer (JS) invited to confirm the accuracy of the entered data from the study. All outcomes of interest were entered into separate sheets. For postdischarge data, if a study recorded more than one time point of data during its followup, the data point with the longest followup time was recorded. This rule was not applicable to the discharge location data, as the first discharge location was of primary interest. If the study used multiple frailty assessment tools, the outcomes measured from each assessment tool were treated as separate data points. For GFR, both the estimated GFR (eGFR) and GFR data were extracted from the study.

In total, 19 outcomes were extracted: three continuous variables and 16 categorical variables. Continuous data included LOS (days), HbA1c (mmol/mol), and GFR (ml/min). The mean, standard deviation (SD), and sample size of both frail and non-frail groups were extracted for continuous outcomes. Data with only interquartile range (IQR) values were converted to SD using the formula SD = IQR/1.35. Categorical data included in-hospital mortality, post discharge mortality, extended stay, rehospitalization, discharge location, postoperative myocardial infarction, cerebrovascular accident (CVA) and stroke, postoperative renal failure, smoking history, hypertension, CVD, PVD, diabetes, COPD, CKD, and cognitive impairment or delirium. The number of patients with the outcomes and the sample size of both frail and non-frail groups were extracted for categorical outcomes. All categorical data were bivariate.

Characteristics of the included studies and their JBI study quality scores are extracted and summarized in Table 1 in the Section “Results.” The following characteristics were included: study publication year, country where the study was conducted, study design, mean age of the study population, size of the study population, number of males in the study, percentage of males, surgical procedure included, frailty assessment tool used, study outcomes of interest, and JBI score. Different frailty scores were expected to be used in different studies selected. The cutoff points for frailty are different between scores but identifications are all based on the Fired Criteria. The essence of identifying frailty is the detection on whether any or more than half of the five frailty phenotype have been showed on patient assessed. These phenotypes might be divided into subcategories or be summarized into specific characteristics while using, for example, the CFS summarized the level of each phenotype into characteristics from “very fir” to “terminally ill” but are comparable among different scores and models meaning that one patient identified as “frail” using one tool are more likely to be identified as “frail” in another tool. Therefore, as long as the method and tools used were kept consistent within the study, the use of these tools were considered valid. The type of the tool and the cutoff point for the tool were then recorded in the study characteristics table.

**TABLE 1:** Characteristics of included studies.

References	Country	Design	Mean age	Population size	Number of males	Male ratio in %	Surgical procedure	Frailty assessment tool (frailty threshold score)	Outcomes	JBI quality assessment score (>5)	JBI total score
1. Aitken et al. (29)	Australia	Retrospective cohort	82	9,752	5,899	60.5	Lower limb arterial, endovascular; lower limb arterial, open; endovascular aortoiliac, open aortoiliac, endovascular carotid, open carotid	HFRS (>5)	Primary: mortality at 30 days and 2 years after index admission. Secondary: LOS, emergency readmission	7	11
2. Al Shakarchi et al. (30)	UK	Retrospective cohort	73.5	184	166	90.2	Elective open abdominal aortic aneurysm (AAA)	CFS (>5)	Primary: 30 days mortality. Secondary: reoperation within 30 days, LOS, readmission within 30 days after discharge.	10	11
3. Ali et al. (31)	USA	Retrospective cohort	67.9	4,704	3,007	53.9	Infrainguinal arterial bypass surgery	mFI-11 (≥0.25)	Primary: 30 days mortality. Secondary: postop myocardial infarction, stroke, renal failure, graft failure	8	11
4. Arya et al. (32)	USA	Retrospective cohort	69.7	15,843	10,262	64.8	Endovascular AR, open AAA repair, infrainguinal bypass, suprainguinal bypass, peripheral EV intervention, carotid stenting, CEA.	mFI-11 (≥0.25)	Primary: Non-home discharge location. Secondary: 30 days morbidity and mortality, LOS	10	11
5. Ayyash et al. (33)	UK	Prospective cohort	72	97	81	84.0	Open abdominal aortic aneurysm repair, endovascular abdominal aortic aneurysm repair, aorto-bifernal graft, fern-fern crossover graft, femoro-popliteal bypass, internal iliac artery repair.	CFS (>5)	30-day readmission, in-hospital mortality, major morbidity, length of stay, perioperative critical care admission.	8	11
6. D’cruz et al. (34)	Singapore	Retrospective cohort	67.8	211	N/A	N/A	Asian population with critical limb ischemia (CLI) who undergo major lower extremity amputation (LEA) including below-knee amputations (BKAs) or above knee amputations (AKAs).	mFI-11 (≥0.25)	Primary: mortality rate at 30 days 6 months, 12 months post-operation, perioperative complications. Secondary: define risk factors of these outcomes.	11	11
7. Donald et al. (35)	USA	Retrospective cohort	69.9	134	94	70.1	Elective open abdominal aortic aneurysm repair, endovascular aneurysm repair, thoracic endovascular aortic repair, suprainguinal bypass, infrainguinal bypass, carotid endarterectomy, peripheral vascular intervention.	CFS (≥5)	Primary: Post surgery discharge to non-home location, mortality at 30 days after surgery. Secondary: readmission, emergency visit within 30 days after discharge, LOS.	11	11
8. Fang et al. (36)	USA	Retrospective cohort	59	379	243	64	All patients (>18 years old) who underwent transtibial or transfemoral amputations at a multihospital academic institution between December 2010 and March 2015	mFI-5 (>2)	Primary: 30 days and 1 year mortality. Secondary: 30 days readmission, unplanned revision, composite adverse events.	9	11
9. Ghaffarian et al. (37)	USA	Retrospective cohort	60.2	415	110	65.9	Open and endovascular aortic aneurysm, carotid endarterectomy, peripheral bypass procedures, peripheral vascular intervention, amputations, haemodialysis access creation, haemodialysis access intervention	CFS (≥5)	All-cause mortality at 1 year and 2 years follow-up post-surgery.	10	11
10. Gilbertson et al. (38)	USA	Retrospective cohort	67.8	163	115	70.0	All patients with endovascular abdominal aortic aneurysm [AAA] repair, thoracic endovascular aortic repair, suprainguinal and infrainguinal bypass, peripheral vascular intervention, carotid endarterectomy, and open AAA recorded in the vascular surgery clinic at the University of Utah Cardiovascular Centre between January 2016 and July 2018	CFS (≥5)	Any major complications, non-home living status, or death within 1 year after vascular surgery	9	11
11. Green et al. (39)	USA	Prospective cohort	86.2	159	79	49.7	Severe aortic stenosis who received transcatheter aortic valve replacement.	Frailty score derived with the markers of frailty (>5)	In-hospital life-threatening or major bleeding, major vascular complication, in-hospital major stroke, in-hospital acute kidney injury, and 30-day mortality.	10	11
12. Iliadis et al. (40)	Germany	Retrospective cohort	78	229	126	55.0	Adults undergoing transcatheter mitral valve repair using Mitra Clip system.	Fried criteria (≥3)	MVARC major or minor bleeding, acute kidney injury requiring haemodialysis, infection, haemodynamic instability/arrhythmias, respiratory failure, total LOS, stay on regular ward and stay on intensive/intermediate care unit	8	11
13. Karam et al. (41)	USA	Retrospective cohort	68	67,308	41,799	62.1	Carotid endarterectomy, lower extremity revascularisation, endovascular abdominal aortic aneurysm repair, open repair of abdominal aortic aneurysm, major lower extremity amputation.	mFI-11 (>0.27)	Primary: 30 days mortality. Secondary: Surgical site infection, occurrence, myocardial infection, Calvin IV complication	10	11
14. Karim et al. (42)	USA	Retrospective cohort	68.3	1,414,080	880,195	62.2	Patient with limb-threatening ischemia who underwent endovascular revascularisation, surgical revascularisation, and no revascularisation	CLTI-FRS (>0.40)	Primary: In-hospital mortality and major complications. Secondary: hospital cost, LOS, in-hospital complications	8	11
15. Khan et al. (43)	USA	Retrospective cohort	73.2	17,983	11,443	63.6	All patients who underwent Trans carotid artery revascularisation (TCAR) from November 2016 to April 2021 in the Vascular Quality Initiative (VQI) Database	mFI-5 (≥0.6)	Primary: in-hospital mortality, extended length of postoperative stay (>1 day), non-home discharge. Secondary: in-hospital stroke, transient ischemic attach, myocardial infarction	11	11
16. Li et al. (44)	China	Prospective cohort	80	146	114	78.1	Patients aged over 65 years old who are diagnosed with T2DM including those with PAD undergoing amputation (stayed for >3 days).	FRAIL scale (≥3)	Primary: prevalence of frailty among older patients with diabetes. Secondary: physical function, diabetic vascular complication, hospitalization, and mortality	8	11
17. Maltese et al. (45)	UK	Prospective cohort	63.3	76	61	80.3	Patient with diabetic foot ulcer including those who underwent amputation.	FI (>0.25)	Primary: Non-healing of DFU at 6 months. Secondary outcome: re-hospitalization at 6-month post index discharge	8	11
18. Panayi et al. (46)	USA	Retrospective cohort	N/A	3,795	2,585	68.1	Head and neck microvascular reconstruction	mFI-5 (≥2)	Primary: LOS, complications and discharge destination, unplanned readmission, mortality within 30 days, occurrence of reopen, surgical, and medical complication.	10	11
19. Partridge et al. (47)	UK	Prospective cohort	76.3	125	86	68.8	Patients aged over 60 years undergoing elective and emergency arterial vascular surgery: lower limb bypass graft, EVAR, open AAA repair, toe/foot amputation, BKA/TKA, AKA, pseudoaneurysm repair, evacuation haematoma.	EFS (≥6.5)	Primary: LOS. Secondary: Postoperative morbidity (medical and surgical complications)	8	11
20. Sareh et al. (48)	USA	Retrospective cohort	61.8	302,798	210,142	69.4	Minor lower extremity amputation	ICD-9 codes (deemed frail if they had ICD-9 diagnostic codes associated with the ten clusters previously defined by the Johns Hopkins Adjusted Clinical Group)	Primary: All-cause readmission within 90 days. Secondary: the need for any re-amputation, including minor and major, in-hospital mortality, and cumulative hospital costs within 90 days of index discharge.	9	11
21. Sarkar et al. (49)	USA	Retrospective cohort	65.6	53	27	50.9	Revascularisation for acute limb ischemia	mFI-11 (≥3)	In-hospital mortality, major amputation, site of discharge, ambulatory statis at follow-up	9	11
22. Thillainadesan et al. (50)	Australia	Prospective cohort	79.5	150	102	68.0	Acute hospitalized older vascular surgery patients underwent both open and endovascular procedures.	Rockwood FI (>0.25) and CFS (>5)	Primary: Hospital acquired geriatric syndromes, delirium, functional decline, complications, pressure injury, fall. Secondary: LOS, medical and surgical complications, discharge disposition, decline in mobility, in hospital and 30 days post discharge mortality, 28 days unplanned readmission, high-risk prescribing at discharge	7	11
23. Tse et al. (51)	USA	Retrospective cohort	66.4	47,197	46,672	98.9	Lower extremity amputation	RAI (>25)	Primary: 30 days mortality. Secondary: postoperative complications, LOS.	8	11
24. Visser et al. (52)	Netherlands	Prospective cohort	72.1	630	486	77.1	Carotid surgery, open aortic surgery, endovascular procedures, peripheral bypass surgery, amputation surgery	GFI (≥4)	Primary: 30 days morbidity. Secondary: 30 days mortality, hospital readmission, type of care facility after discharge	11	11

HFRS, hospital frailty risk score; AVFS, addenbrooke’s vascular frailty score; CFS, clinical frailty scale; CLTI-FRS, CLTI frailty risk score; ED-5D, euroqol-5 dimension-5 levels; EFS, edmonton frail scale; RAI, risk analysis index; GFI, groningen frailty indicator; KCCQ, kansas city cardiomyopathy questionnaire; mFI, modified frailty index; mFI-5, 5-items modified frailty index; TAVI, transfemoral aortic valve implantation; TCAR, trans carotid artery revascularization.

Subgroups were based on the follow-up time, characteristics of the study population, and therapy characteristics. Information was retrieved from each included study and extracted manually by the main reviewer. The subgroups included the length of total follow-up (1 = 30 days and less, 2 = more than 30 days), the mean age of the study population (1 = less than 70 years old, 2 = 70 years old and more), surgery type involved (1 = vascular surgeries, 2 = only amputation was involved), severity of the disease (1 = mild, 2 = severe), amputation and foot ulcer history (0 = no, 1 = yes), polymedication (0 = no, 1 = yes), and wound type (1 = open wound, 2 = other types of wound). While identifying subgroups, the identification criteria were adapted based on the characteristics of selected studies and kept consistent throughout the subgroup identification process to achieve as good and accurate subgroup identification as possible. Subgroup in mean age was defined utilizing the 70 years old cutoff value proposed in Jacobs’s study (28) to best suit the age profile of the relevant studies. As medication regimes are not mentioned in most of the selected studies, we have identified the “polymedication” group as studies that have mentioned that their patients were taking more than one kind of medication. Studies that did not specify their medication regime or specify that their patient has only taken one medication were classified as the “no polymedication” subgroup. Regarding wound types, studies covering only open surgeries or amputations were identified as “open wounds,” and studies covering mixed types of surgeries and endovascular surgeries only were identified as “other wounds.”

### Statistical analysis

Data analysis was conducted by one researcher (JS) using Comprehensive Meta-Analysis software version 3. Each outcome was analyzed separately. Due to the heterogenicity of each outcome, a random-effects model was used to describe the overall effect size of the data. Biased studies were removed from the analysis. The standardized mean difference (MD) and 95% confidence interval were determined for continuous outcomes, including LOS, HbA1c, and eGFR values. RR and 95% confidence interval were determined for each categorical outcome, including in-hospital mortality, postdischarge mortality, rehospitalization, discharge location, extended hospital stay, postoperative myocardial infarction, postoperative renal failure, CVA and stroke, smoking history, hypertension, CVD, PVD, diabetes, COPD, CKD, and cognitive impairment and delirium. The heterogeneity test was conducted to determine the sample heterogeneity between studies, with *I*^2^ > 50% or *p* < 0.05 indicating significant heterogeneity. The results were drafted as forest plots.

A subgroup analysis was conducted for postdischarge mortality, length of hospital stays, and rehospitalization. This was done based on the following subgroups to compare the difference between the groups relating: the length of follow-up (30 days or less vs. more than 30 days), mean age (less than 70 years old vs. 70 years or more), surgery type (vascular surgery vs. amputation), severity (mild vs. severe), existence of amputation and foot ulcer history (yes vs. no), whether polymedication regime was mentioned (yes vs. no) and the type of wound (Open wound vs. others).

Publication bias of each study was assessed using Egger’s regression analysis, with *p* < 0.05 indicating significant publication bias and *p* ≥ 0.05 indicating nonsignificant publication bias. Analyses were conducted both before and after removing the biased studies to examine whether the significance of a certain outcome would remain significant after removing biased studies. Funnel plots were used to visualize publication bias. An asymmetrical funnel plot indicated possible publication bias, and a symmetrical funnel indicated no significant publication bias.

## Results

### Study selection: PRISMA

**FIGURE 1:**
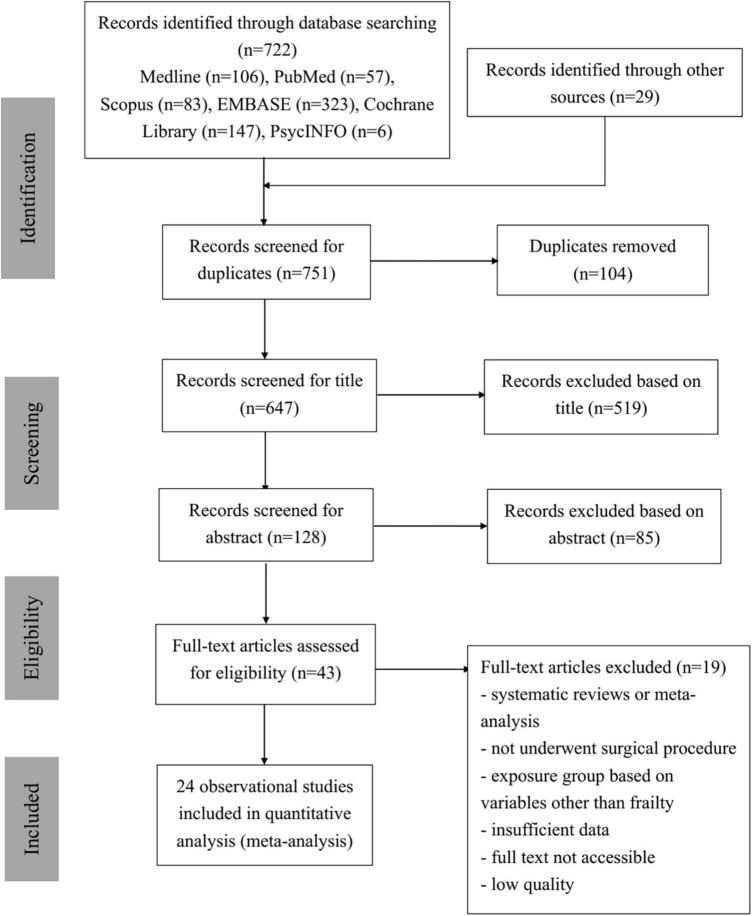
PRISMA flowchart.

As shown in Figure 1, 722 studies were collected by the two reviewers from six different databases—Medline, PubMed, Scopus, EMBASE, Cochrane Library, and APA PsycINFO—with 29 additional studies from other resources. In total of 751 identified studies were imported into EndNote, and 104 duplications were removed. The remaining 647 articles were then screened for relevance and eligibility based on their titles and abstracts. After the titles and abstracts were screened, 85 articles were excluded due to not being eligible. The remaining 43 relevant articles then underwent full-text screening and quality assessment. Nineteen studies were excluded due to one or more of the following reasons: studies were systematic reviews or meta-analyses, participants did not undergo any surgical procedures, exposure groups were grouped based on factors other than frailty scores, data were insufficient in the study, full text was not accessible online, or studies were of low quality with JBI scores less than 5. Thus, 24 observational studies were included in the quantitative data analysis, and their study characteristics are displayed in Table 1. Eventually, 24 studies contributed to the results of this analysis (29–52).

### Study characteristics

Detailed study characteristics of the included studies and their JBI quality scores are displayed in Table 1. In total, 1,886,611 participants were included in the 24 included observational studies (29–52). All 24 studies were cohort studies using either prospective or retrospective data collection methods. The included studies were conducted in Australia (29, 50), the United States (31, 32, 35–39, 41–43, 46, 48, 49, 51), the United Kingdom (30, 33, 45, 47), China (44), Germany (40), Singapore (34), and the Netherlands (52). Among the 24 included studies, five (33, 42, 43, 48, 50) investigated in-hospital mortality and provided unbiased data; 12 (29–31, 34, 36, 38, 39, 44, 49–51, 53) investigated postdischarge mortality; nine (29, 30, 33, 34, 36, 38, 46, 48, 50) investigated rehospitalization number; and five (32, 38, 43, 46, 50) investigated discharge locations. For additional outcomes, each outcome has multiple studies contributing to data ranging from five to 18 studies. Five studies (34, 44, 45, 48, 51) investigated patients who underwent amputations only, and the remaining studies investigated patients who underwent different types of vascular surgeries, including elective open abdominal aortic aneurysm repair, endovascular aneurysm repair, thoracic endovascular aortic repair, suprainguinal bypass, infrainguinal bypass, carotid endarterectomy, peripheral vascular intervention, transcatheter mitral valve repair, carotid endarterectomy, lower extremity revascularization, and major lower extremity amputation.

### Frailty score

Different validated frailty scores were used in the included studies to assess the participants’ levels of frailty. Among the 24 studies, six (30, 31, 35, 37, 38, 50) utilized the CFS, which identifies individuals scoring 5 or higher as frail. 3 studies (36, 43, 46) utilized the five-points version of the MFI (mFI-5), which identifies individuals scoring 2 and higher as frail; and five studies (32–34, 41, 49) utilized the 11-items version of the MFI (mFI-11), which identifies individuals scoring 0.25 or 0.27 and higher as frail. Two studies utilized the Rockwood Frailty Index, which defines individuals scoring higher than 0.25 as frail, and used the CFS as a separate assessment (45, 50). The remaining nine studies all utilized different frailty score (29, 39, 40, 42, 44, 47, 48, 51, 52). Aitken et al. (29) utilized the HFRS to identify individuals scoring greater than 5 as frail. Green et al. (39) utilized a frailty score derived from markers of frailty that identify individuals scoring higher than 5 as frail. Iliadis et al. (40) utilized the Fried Criteria, which identifies individuals scoring 3 or higher as frail. Karim et al. (42) utilized the Chronic limb-threatening ischemia (CLTI) Frailty Risk Score, which identifies individuals scoring higher than 0.4 as frail. Li et al. (44) utilized the FRAIL scale, which identifies individuals scoring 3 and higher as frail. Partridge et al. (47) utilized the Edmonton Frail Scale (EFS), which identifies individuals scoring 6.5 or higher as frail. Sareh et al. (48) utilized the ICD-9 codes, which deem individuals frail if they have any ICD-9 diagnostic codes associated with the 10 clusters previously defined by the Johns Hopkins Adjusted Clinical Group. Tse et al. (51) utilized the Risk Analysis Index (RAI), which identifies individuals scoring higher than 25 as frail. Finally, Visser et al. (52) utilized the Groningen Frailty Indicator (GFI), which identifies individuals scoring 4 and higher as frail.

### In-hospital mortality and postdischarge mortality

**FIGURE 2:**
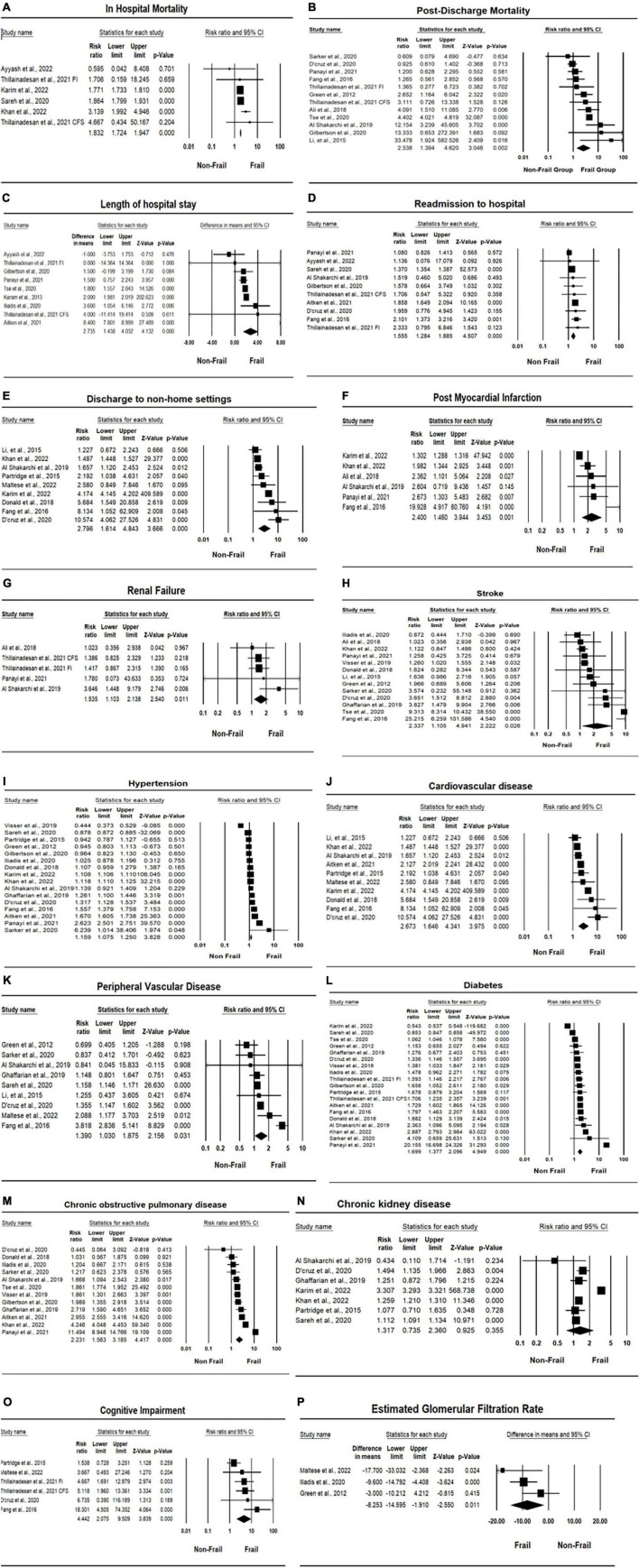
Forest plots of association between frailty and health outcomes for patients who underwent vascular surgery. **(A)** In-hospital mortality. **(B)** Post-discharge mortality. **(C)** Length of hospital stay. **(D)** Rehospitalisation. **(E)** Discharge location. **(F)** Post-operative myocardial infarction. **(G)** Renal failure. **(H)** Stroke. **(I)** Hypertension. **(J)** Cardiovascular disease (CVD). **(K)** Peripheral vascular disease (PVD). **(L)** Diabetes. **(M)** Chronic obstructive pulmonary disease (COPD). **(N)** Chronic kidney disease (CKD). **(O)** Cognitive impairment. **(P)** Estimated glomerular filtration rate (eGFR).

Five studies (33, 42, 43, 48, 50) reported in-hospital mortality for both the frail and non-frail groups, with two (42, 43) of those studies reporting a significant difference in in-hospital mortality (*p* < 0.001). Overall, there was a significant difference in in-hospital mortality between the frail and non-frail groups, with a risk ratio (RR) of 1.832 and a significant *I*^2^ result (CI: 1.724, 1.947, *p* < 0.001, *I*^2^ = 60.943, *p I*^2^ = 0.025). The forest plot displayed in Figures 2A–D indicates that the frail group had significantly higher in-hospital mortality. As for postdischarge mortality, the length of follow-up was either within 30 days after index discharge or within 2 years after discharge. Twelve studies reported postdischarge mortality, with five of those studies (30, 31, 46, 50, 51) reporting mortality within 30 days after index discharge, producing 6 data points, and six studies (34, 36, 38, 39, 44, 49) reporting mortality more than 30 days after discharge. Overall, the difference between the frail group and non-frail group in postdischarge mortality was significant. The RR was 2.358, with a significant *I*^2^ result (CI: 1.384, 4.620, *p* = 0.002, *I*^2^ = 86.960, *p I*^2^ < 0.001). The RR and heterogenicity test results of in-hospital mortality and postdischarge mortality are summarized in Table 2. The forest plot displayed in Figures 2A–D indicates that the frail group had significantly higher postdischarge mortality.

**TABLE 2:** Standardized mean difference and risk ratio on categorical outcomes.

Categorical outcomes	Studies (n)	Participants (N)	Risk ratio (95% CI)	*P-*value	*Q-*test	*I*^2^ (%)	*P*-value
In-hospital mortality	6	1,735,108	1.832 (1.724, 1.947)	<0.001	12.802*	60.943*	0.025
Postdischarge mortality	12	57,141	2.358 (1.394, 4.620)	0.002	84.355***	86.960***	<0.001
Rehospitalisation	10	317,529	1.555 (1.284, 1.885)	<0.001	33.402	73.055***	<0.001
Discharge location	6	37,934	2.453 (1.804, 3.335)	<0.001	47.336***	89.437***	<0.001
Postoperative myocardial infarction	6	1,441,125	2.400 (1.460, 3.944)	0.001	26.394***	81.056***	<0.001
Postoperative renal failure	5	8,833	1.535 (1.103, 2.138)	0.011	4.201	4.778	0.380
CVA and stroke	13	76,035	2.337 (1.105, 4.941)	0.026	452.821***	97.350***	<0.001
Hypertension	16	1,760,842	1.159 (1.075, 1.250)	<0.001	5027.156***	99.702***	<0.001
Cardiovascular disease	10	1,443,070	2.673 (1.646, 4.341)	<0.001	6037.084***	99.851***	<0.001
Peripheral vascular disease	9	304,421	1.390 (1.030, 1.875)	0.031	73.303***	89.086***	<0.001
Diabetes	19	1,798,437	1.699 (1.377, 2.096)	<0.001	14982.507***	99.880***	<0.001
Chronic obstructive pulmonary disease	12	80,746	2.231 (1.563, 3.185)	<0.001	737.227***	98.508***	<0.001
Chronic kidney disease	7	1,735,796	1.317 (0.735, 2.360)	0.355	14168.822***	99.958***	<0.001
Cognitive impairment	6	941	4.442 (2.075, 9.509)	<0.001	11.081*	54.878*	0.050

CI, confidence interval. *p < 0.05, **p < 0.01, ***p < 0.001.

The results of the subgroup analysis for postdischarge mortality are summarized in Table 3. Postdischarge mortality was significantly higher in the frail group in articles with shorter postoperative follow-up (within 30 days after index discharge) (RR = 3.189, CI: 1.661, 6.126, p < 0.001, I^2^ = 74.637, p I^2^ < 0.001), with higher age (RR = 4.816, CI: 1.857, 12.494, p = 0.001, I^2^ = 47.465), with mild severity (RR = 3.198, CI: 1.453, 7.037, *p* = 0.004, *I*^2^ = 88.222, *p I*^2^ < 0.001), and with open wounds (RR = 2.851, CI: 1.264, 6.428, *p* = 0.012, *I*^2^ = 89.923, *p I*^2^ < 0.001). The subgroup analysis results also showed that in patients with no foot ulcer and amputation history, the difference in postdischarge mortality between frail and non-frail patients was significant (RR = 3.213, CI: 1.582, 6.524, *p* = 0.001, *I*^2^ = 56.297, *p I*^2^ = 0.033), but the difference is not significant in patients having amputation and foot ulcer history. Postdischarge mortality was significantly higher in the frail group for participants who underwent all types of vascular surgeries (RR = 2.150, CI: 1.378, 4.571, *p* = 0.003, *I*^2^ = 52.542, *p I*^2^ = 0.032), but no significant difference was found between frail and non-frail patients in articles that studied amputation patients only (RR = 3.585, CI: 0.768, 16.721, *p* = 0.104, *I*^2^ = 93.573, *p I*^2^ < 0.001). The subgroup analysis also indicated that the difference between vascular surgery patients and amputation patients was not significant (*p*_*between*_ = 0.673). The heterogenicity still remains high for studies within each subgroup.

**TABLE 3:** Subgroup analysis on the association between frailty score and postdischarge mortality.

	Studies (n)	Risk ratio (95% CI)	*P-*value	*Q*-test	*I*^2^ (%)	*P* between
**Length of follow-up**						
≤30 days	6	3.189 (1.661, 6.126)	<0.001	19.713	74.637**	0.227
>30 days	6	1.737 (0.828, 3.641)	0.144	13.149	61.974*	
**Mean age**						
<70 years old	6	2.219 (0.877, 5.616)	0.092	41.467	87.942***	0.061
70 years old and more	5	4.816 (1.857, 12.494)	0.001	7.614	47.465	
**Surgery type**						
Vascular surgeries	9	2.150 (1.378, 4.571)	0.003	16.857	52.542*	0.673
Amputation only	3	3.585 (0.768, 16.721)	0.104	31.123	93.574***	
**Severity**						
Mild	8	3.198 (1.453, 7.037)	0.004	59.432	88.222***	0.581
Severe	4	2.379 (1.189, 4.762)	0.014	2.307	<0.001	
**Amputation and foot ulcer history**						
No	7	3.213 (1.582, 6.524)	0.001	13.729	56.297*	0.569
Yes	5	2.170 (0.688, 6.843)	0.186	43.116	90.723***	
**Poly-medication**						
No	11	2.993 (1.561, 5.738)	0.001	56.787	82.390***	0.125
Yes	1	1.284 (0.542, 3.044)	0.570	<0.001	<0.001	
**Wound**						
Open wound	7	2.851 (1.264, 6.428)	0.012	59.543	89.923***	0.966
Others	5	2.920 (1.427, 5.976)	0.003	4.118	2.875	

CI, confidence interval. *p < 0.05, **p < 0.01, ***p < 0.001.

### Frailty and length of hospital stay

Eight studies (29, 33, 38, 40, 46, 50, 51) reported the length of hospital stays for patients who underwent vascular surgery, producing nine data points. Four studies (29, 40, 46, 50) reported significant differences in LOS between frail and non-frail groups, with three studies (29, 40, 46) reporting *p* < 0.001 and one study (50) reporting *p* = 0.003. Overall, the length of hospital stay was significantly higher in frail groups compared to non-frail groups, with a mean difference of 2.735 (CI: 1.438, 4.032, *p* < 0.001, *I*^2^ = 98.219, *p I*^2^ < 0.001) and an effect size of 0.274 (CI: 0.135, 0.414, *p* < 0.001, *I*^2^ = 98.970, *p I*^2^ < 0.001). Detailed results are summarized in Table 4. The forest plot for the length of hospital stay displayed in Figures 2A–D further indicates that the frail group had a longer length of hospital stay.

**TABLE 4:** Standardized mean difference and effect size on length of stay and eGFR.

Continuous outcomes	Studies (n)	Participants (N)	Mean difference (95% CI)	MD *p-*value	MD *Q*-test	MD*I*^2^ (%)	*P-*value	Standardized mean difference (95% CI)	SMD *p-*value	SMD *Q*-test	SMD*I*^2^ (%)	*P-*value
Length of stay	9	128,691	2.735 (1.438, 4.032)	<0.001	499.297	98.219	<0.001	0.274 (0.135, 0.414)	<0.001	776.922	98.970	<0.001
GFR/eGFR	3	464	−8.253 (−14.595, −1.910)	0.011	3.748	46.634	0.154	−0.370 (−0.633, −0.106)	0.006	3.579	44.119	0.167

CI, confidence interval.

The results for the MDs for the length of hospital stays are summarized in Table 5. The subgroup analysis for whether patients were treated with a polymedication regime or not was not performed for the length of hospital stay outcome, as not all studies reporting length of hospital stay used a polymedication regime. The results indicated that the difference in LOS between the frail and non-frail groups was not significant with the difference in follow-up time (*p*_*between*_ = 0.292), mean age (*p*_*between*_ = 0.431), whether patients underwent all kinds of vascular surgery or amputation only (*p*_*between*_ = 0.506), whether a history of amputation and foot ulcer was present (*p*_*between*_ = 0.669), and the types of wounds (*p*_*between*_ = 0.637). However, the difference in length of hospital stay between frail and non-frail groups was significant between mild and severe severity (*p*_*between*_ = 0.009), with the mild severity subgroup showing significantly longer LOS in frail patients (RR = 3.151, CI: 1.774, 4.528, *p* < 0.001, *I*^2^ = 98.875, *p I*^2^ < 0.001; effect size = 0.327, CI: 0.175, 0.480, *p* < 0.001, *I*^2^ = 99.349, *p I*^2^ < 0.001).

**TABLE 5:** Subgroup analysis on the association between frailty score and length of hospital stay.

	Studies (n)	Mean difference (95% CI)	*P-*value	*Q*-test	*I*^2^ (%)	*P* between	Effect size (95% CI)	*P-*value	*Q*-test	*I*^2^ (%)	*P* between
**Length of follow-up**											
≤30 days	6	1.861 (1.619, 2.104)	<0.001	9.015	44.537	0.292	0.181 (−0.001, 0.363)	0.051	718.021	99.304***	**0.020**
>30 days	3	4.559 (−0.450, 9.568)	0.074	65.562	96.949***		0.471 (0.307, 0.634)	<**0**.**001**	3.921	48.988	
**Mean age**											
<70 years old	3	1.949 (1.806, 2.092)	<**0**.**001**	2.919	31.493	0.431	0.283 (0.052, 0.514)	**0.016**	684.084	99.708***	0.630
70 years old and more	5	3.462 (−1.725, 8.649)	0.191	54.892	92.713***		0.257 (−0.025, 0.540)	0.074	19.311	79.287**	
**Surgery type**											
Vascular surgeries	8	2.705 (0.048, 5.362)	**0**.**046**	446.545	98.432***	0.506	0.327 (0.214, 0.439)	<**0**.**001**	88.563	92.096***	**0**.**002**
Amputation only	1	1.800 (1.557, 2.043)	**<0**.**001**	<0.001	<0.001		0.144 (0.125, 0.164)	<**0**.**001**	<0.001	<0.001	
**Severity**											
Mild	6	3.151 (1.774, 4.528)	<**0**.**001**	444.579	98.875***	**0.009**	0.327 (0.175, 0.480)	<**0**.**001**	767.586	99.349***	**0.043**
Severe	3	−0.816 (−3.479, 1.846)	0.548	0.405	<0.001		0.023 (−0.228, 0.275)	0.856	0.730	<0.001	
**Amputation and foot ulcer history**											
No	7	2.770 (−1.040, 6.580)	0.154	244.656	97.548***	0.669	0.249 (0.024, 0.475)	**0.030**	75.507	92.054***	0.873
Yes	2	1.938 (1.757, 2.119)	<**0**.**001**	2.588	61.366		0.277 (0.017, 0.538)	**0.037**	683.803	99.854***	
**Wound**											
Open wound	3	1.775 (1.379, 2.172)	<**0**.**001**	2.532	21.018	0.637	0.163 (0.106, 0.220)	<**0**.**001**	3.216	37.815	**0.003**
Others	6	2.728 (−1.211, 6.667)	0.175	443.256	98.872***		0.376 (0.249, 0.503)	<**0**.**001**	60.746	91.769***	

CI, confidence interval, no medication subgroups data due to all studies belong to no ploy-medication group. **p < 0.01, ***p < 0.001. The bold values mean significant *p*-values.

Subgroup analysis further showed that frailty is more strongly associated with length of hospital stay in articles studied vascular surgery patients (effect size = 0.327, CI: 0.214, 0.439). The association was similar between articles studied patients with amputation and foot ulcer history (effect size = 0.249, CI: 0.024, 0.475, *I*^2^ = 97.548) and patients without amputations and foot ulcer history (effect size = 0.277, CI: 0.017, 0.538, *I*^2^ = 61.366). The heterogenicity still remains high for studies within the two subgroups (Table 5).

### Frailty and rehospitalization rate

Nine studies (29, 30, 33, 34, 36, 38, 46, 48, 50) reported rehospitalization rates for patients who underwent vascular surgery. Three of these studies (29, 36, 46) reported significantly higher rehospitalization rates in the frail groups, with p-values less than or equal to 0.001. Overall, rehospitalization rates were higher in frail patients after index discharge compared to non-frail patients, with an RR of 1.555 (CI: 1.284, 1.885, *p* < 0.001, *I*^2^ = 73.055). Detailed results are summarized in Table 2. The forest plot for rehospitalization displayed in Figures 2A–D further indicates that the frail group had higher rehospitalization rates.

The subgroup analysis results for rehospitalization are summarized in Table 6. The subgroup analysis regarding whether patients were treated with a polymedication regime was not done for the rehospitalization outcome because all studies reporting rehospitalisation did not use a polymedication regime. The result indicated that the difference in rehospitalisation between the frail and non-frail groups was more significant in articles with longer length of follow-up time (more than 30 days after index discharge) (RR = 1.685, CI: 1.320, 2.152, *p* < 0.001, *I*^2^ = 86.298, *p*_*between*_ = 0.038), higher mean age (>70 years old) (RR = 1.856, CI: 1.651, 2.088, *p* < 0.001, *I*^2^ < 0.001, *p*_*between*_ = 0.001), and patients with other types of wounds (RR = 1.854, CI: 1.650, 2.084, *p* < 0.001, *I*^2^ < 0.001, *p*_*between*_ = 0.019).

**TABLE 6:** Subgroup analysis on the association between frailty score and rehospitalisation rate.

	Studies (n)	Risk ratio (95% CI)	*P-*value	*Q*-test	*I*^2^ (%)	*P* between
**Length of follow-up**						
≤30 days	5	1.167 (0.911, 1.494)	0.221	2.524	<0.001	**0.038**
>30 days	5	1.685 (1.320, 2.152)	<**0**.**001**	29.192	86.298***	
**Mean age**						
<70 years old	4	1.558 (1.216, 1.998)	<**0**.**001**	4.544	33.986	**0.001**
70 years old and more	5	1.856 (1.651, 2.088)	<**0**.**001**	0.429	<0.001	
**Surgery type**						
Vascular surgeries	8	1.622 (1.246, 2.112)	<**0**.**001**	14.476	51.644*	0.211
Amputation only	2	1.371 (1.355, 1.387)	<**0**.**001**	0.572	<0.001	
**Severity**						
Mild	7	1.537 (1.252, 1.887)	<**0**.**001**	32.316	81.433***	0.570
Severe	3	1.926 (0.909, 4.082)	0.087	0.311	<0.001	
**Amputation and foot ulcer history**						
No	7	1.532 (1.114, 2.108)	**0.009**	13.578	55.811*	0.818
Yes	3	1.617 (1.162, 2.249)	**0.004**	4.443	84.990	
**Wound**						
Open wound	5	1.401 (1.143, 1.717)	**0.001**	7.494	46.623	**0.019**
Others	5	1.854 (1.650, 2.084)	<**0.001**	0.456	<0.001	

CI, confidence interval, no medication subgroups data due to all studies belong to no ploy-medication group. *p < 0.05, **p < 0.01, ***p < 0.001. The bold values mean significant *p*-values.

The subgroup analysis result also indicated that, the association between frailty and rehospitalisation is stronger in patients underwent vascular surgeries (RR = 1.622, CI: 1.246, 2.112, *p* < 0.001, *I*^2^ = 51.644), in more severe patients (RR = 1.926, CI: 0.909, 4.082, *p* = 0.087, *I*^2^ < 0.001), and in patients with amputation and foot ulcer history (RR = 1.617, CI: 1.162, 2.249, *p* = 0.004, *I*^2^ = 84.990). However, the difference in rehospitalization between the frail and non-frail groups was not significant between subgroup of patients who underwent all kinds of vascular surgery and patients who underwent amputation only (*p*_*between*_ = 0.211). The difference is also not significant between patients with mild severity and patients with severe severity (*p*_*between*_ = 0.570), and between patients who had a history of amputation and foot ulcers and patients who did not (*p*_*between*_ = 0.818). No further subgroup analysis was done on other outcomes due to lack of studies available in each subgroup.

### Frailty and discharge location

Five studies (32, 38, 43, 46, 50) reported the discharge location for patients who underwent vascular surgery, producing six data points. The types of discharge locations were classified as home location and non-home location, including rehab centers, health care facilities, and other health service institutions. Four studies (32, 38, 43, 46) reported significantly higher non-home discharge in the frail groups, with two studies (43, 46) reporting *p* < 0.001 and two studies (32, 38) reporting *p* < 0.05. Overall, non-home discharge was significantly higher in the frail group, with an RR of 2.453 (CI: 1.804, 3.335, *p* < 0.001, *I*^2^ = 89.437). Detailed results are summarized in Table 2. The forest plot for the discharge location displayed in Figures 2E–H further indicates that the frail group has a higher rate of non-home discharge.

### Comorbidities, chronic conditions, and medical history

Comorbidities, chronic conditions, and medical history investigated in this review include postoperative myocardial infarction, postoperative renal failure, CVA and stroke incidence, hypertension, CVD, PVD, diabetes, COPD, CKD, and cognitive impairment, including postoperative delirium. Overall, the incidences of all investigated chronic conditions and comorbidities were significantly higher in the frail group compared to the non-frail group, except in the case of CKD (*p* = 0.355). Detailed results are summarized in Table 2. Forest plots for chronic conditions and medical history are displayed in Figures 2E–P. One notable outcome among the participants was diabetes. This outcome was reported by most of the included studies (18 studies). The results showed that, at baseline, a significantly higher number of participants in the frail group had diabetes compared to the non-frail group (RR = 1.697, CI: 1.368, 2.105, *p* < 0.001, *I*^2^ = 99.884). The forest plot for diabetes displayed in Figures 2I–L further indicates that the frail group had higher incidences of diabetes at baseline. Another notable outcome was the cognitive impairment associated with the participants. Among the six data points reported, two studies were specifically related to postoperative delirium. The results showed significantly higher incidences of cognitive impairment and postoperative delirium in frail participants who underwent vascular surgery (RR = 4.4442, CI: 2.075, 9.509, *p* < 0.001, *I*^2^ = 54.878). The forest plot for cognitive impairment displayed in Figures 2M–P further indicates that the frail group had higher incidences of cognitive impairment.

### HbA1c and eGFR

HbA1c was originally designed to be investigated with consideration of the high proportion of participants affected by diabetes. However, insufficient data were obtained from the eligible included studies. Thus, the results for HbA1c were missing from this meta-analysis. The eGFR data were collected from studies reporting both eGFR and GFR values. Three studies (39, 40, 45) reported either the eGFR or GFR values of participants who underwent vascular surgery. The estimated MD was −8.253 (CI: −14.595, −1.910, *p* = 0.154, *I*^2^ = 46.634). Overall, eGFR in the frail group was significantly lower than in the non-frail group, with an effect size of −0.370 (CI: −0.633, −0.106, *p* = 0.167, *I*^2^ = 44.119). Detailed results are summarized in Table 4. The forest plot for eGFR displayed in Figures 2M–P further indicates that the frail group had lower eGFR values.

### Publication bias

**FIGURE 3:**
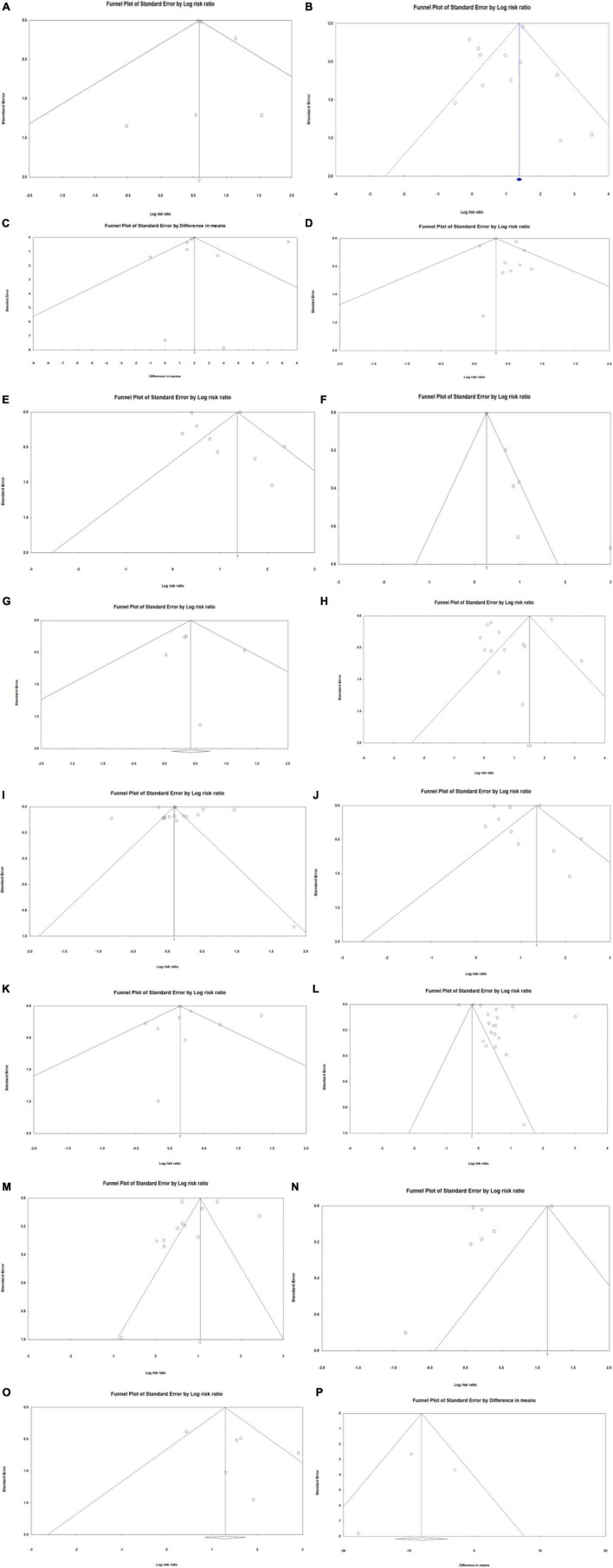
Funnel plots of publication bias investigating association between frailty and health outcomes for patients who underwent vascular surgery. **(A)** In-hospital mortality. **(B)** Post-discharge mortality. **(C)** Length of hospital stay. **(D)** Rehospitalisation. **(E)** Discharge location. **(F)** Post-operative myocardial infarction. **(G)** Renal failure. **(H)** Stroke. **(I)** Hypertension. **(J)** Cardiovascular disease (CVD). **(K)** Peripheral vascular disease (PVD). **(L)** Diabetes. **(M)** Chronic obstructive pulmonary disease (COPD). **(N)** Chronic kidney disease (CKD). **(O)** Cognitive impairment. **(P)** Estimated glomerular filtration rate (eGFR).

The results of publication bias determined through Egger’s regression analysis are displayed in Table 7. The results for all outcomes were nonsignificant, except for postoperative myocardial infarction (*p* = 0.011). This indicates that there was minimal publication bias among the investigated outcomes, except for postoperative myocardial infarction, for which significant publication bias was found. Funnel plots for each outcome are displayed in Figures 3A–P. As all the plots were symmetrical for all outcomes, including postoperative myocardial infarction, minimal publication bias was likely in all outcomes.

**TABLE 7:** Egger’s regression analysis on publication bias for included studies on categorical outcomes.

Outcomes	t (95% CI)	*P-*value (2-tailed)
In-hospital mortality	0.928 (−1.512, 3.030)	0.406
Post-discharge mortality	1.444 (−3.319, 0.708)	0.179
Length of stay	0.747 (−4.509, 8.674)	0.479
Rehospitalisation	1.308 (−0.635, 2.299)	0.227
Discharge location	0.825 (−31.487, 15.205)	0.437
Postoperative myocardial infarction	4.485 (0.812, 3.449)	0.011
Postoperative renal failure	0.451 (−3.077, 4.092)	0.683
CVA and stroke	1.536 (−8.186, 1.457)	0.153
Hypertension	0.173 (−10.204, 11.994)	0.865
Cardiovascular disease	1.084 (−30.622, 11.043)	0.310
Peripheral vascular disease	1.014 (−1.483, 3.709)	0.344
Diabetes	1.345 (−5.583, 25.218)	0.196
Chronic obstructive pulmonary disease	0.280 (−7.952, 6.177)	0.785
Chronic kidney disease	1.353 (−77.970, 24.205)	0.234
Cognitive impairment	1.280 (−2.261, 6.128)	0.270
GFR/eGFR	0.370 (−37.307, 35.195)	0.774

CI, confidence interval.

## Discussion

### Overall results

This meta-analysis included studies from 2012 to 2022, with the majority of the studies being conducted after 2019 (70%). A growing number of studies focusing on frailty and its association with postoperative health outcomes in vascular surgery patients have indicated a growing potential for development in frailty assessment tools and postoperative care of major vascular surgery patients. The results indicate that, overall, frail patients are significantly likelier to experience mortality, to have a longer hospital LOS, to have a higher chance of discharge to non-home locations, and to be readmitted for hospital treatment after vascular surgeries.

With reduced physiological reserves, frail patients are more vulnerable to postoperative complications. This vulnerability causes them to take a longer time to recover or even makes them unable to recover. It is likelier for secondary admission and other forms of health care to be required after index discharge of frail patients, as indicated by the significantly higher rehospitalization rate and higher non-home discharge rate in the frail group.

### In-hospital mortality and postdischarge mortality

Long-term mortality data were not included in this meta-analysis due to the differences in study follow-up time. The included studies reporting mortality of more than 30 days after index discharge between 60 days and 2 years. Previous studies with larger sample sizes and statistical analyses that considered confounders showed no association between long-term mortality and frailty (54, 55). Long-term mortality was generally low in both the frail and non-frail groups (36, 38, 39, 44, 49). Loss of follow-up and change in chronic conditions can both contribute to the lower number of records in long-term mortalities.

In-hospital mortality was largely impacted by differences in discharge policies between hospitals and differences in the severity of different patient groups; thus, many studies concluded that in-hospital mortality should be considered together with postdischarge mortality (56–60). The results of this meta-analysis showed that both in-hospital mortality and postdischarge mortality were significantly higher in the frail group, indicating that patients with frailty were likelier to experience mortality after vascular surgeries. The frail group was twice as likely to experience both in-hospital mortality and postdischarge mortality compared to the non-frail group. This result aligns with the results of previous studies (61–63) and other observational studies (31, 32, 64–71).

Regardless of the nature of the frailty assessment tool (whether it focuses on risk or independence), the frailty score could reflect the level of vulnerability of the patient to adverse surgical outcomes, for which a higher frailty score is associated with higher mortality. This can be attributed to both the number of comorbidities and population characteristics, such as age and wound types of certain individuals. However, the frailty score alone should not be the reason for eligibility for surgery, as not all frail patients experienced mortality and not all mortality occurred within the frail group. Comorbidities, chronic conditions, medical history, and the type of frailty assessment tool used to assess frailty were all associated with frailty scores and should all be considered when considering one’s eligibility for major surgery.

The subgroup analysis further indicated that risk factors impact the association between frailty and postdischarge mortality among vascular surgery and amputation patients. Mortality within 30 days after index discharge was more significantly associated with frailty, with the frail group being three times likelier to experience frailty within 30 days compared to the non-frail group. The frail group was 1.7 times likelier to experience mortality than non-frail groups in studies followed up for more than 30 days in postdischarge mortality. This result indicates that frailty was more significantly related to short-term mortality than to longer-term mortality. This suggests careers of frail patients would need intensive care even after hospital discharge, especially within the short period following the index discharge.

In terms of age, for participants under 70 years old, the frail group was twice as likely to experience mortality as the non-frail group. However, the RR increased significantly to 4.815 for participants over 70 years of age, indicating a higher risk of mortality with increased age. Is this because aging itself is a risk factor for surgical complications (72–74). Older age is also related to a decline in physiological functions, which makes physiological homeostatic harder to retrieve after experiencing major disturbances such as vascular surgery (47, 73, 75, 76); this often leads to difficulties in wound healing, which is even harder if the wound is open and requires extra care and physiological function to heal (77–80). With these difficulties, mortality is likely to happen in frail patients due to poor wound closing, inflammation, multimorbidity, and other surgical complications (81–83).

The difference in postdischarge mortality between the frail and non-frail groups was more significant in amputation patients compares to patients underwent all other types of vascular surgeries. In vascular surgery patients, frail participants are twice as likely to experience mortality. Among participants undergoing amputation only, frail participants were 3.5 times likely to experience mortality. This result indicates that frail patients are more at risk if they underwent amputation surgeries compare to other vascular surgery types. It can be due to the differences in response to wound healing and postoperative recovery. The difference between the two subgroup is, however, not significant indicated by the insignificant *p*-value. Although some vascular surgery articles have studied multiple vascular surgeries, the heterogenicity within the vascular surgery subgroups is lower than the amputation subgroup (Table 3). This suggests that data are homogeneous among the nine vascular surgery articles, and data are quite different among the three amputation articles. More articles studies on amputation patients are needed in the future meta-analysis work. For vascular surgery patients, the low heterogenicity and significant *p*-value are sufficient to indicate that frail patients are significantly associated with postdischarge mortality. This aligns with previous findings (60, 61).

Patients undergoing amputation were often associated with past or current foot ulcer histories that come with comorbidities to slow down the recovery process. Hence, it is expected that participants with foot ulcer histories would have significantly higher mortality compared to participants without foot ulcer histories. Nevertheless, subgroup analysis results of foot ulcer history did not agree with this hypothesis. For participants without amputation or foot ulcer histories, the frail group was three times likelier to experience mortality compared to the non-frail group. For participants with amputation and foot ulcer histories, the frail group was only twice as likely to experience mortality as the non-frail group. The lower RR value can be contributed by the higher heterogenicity among articles with patients having amputation and foot ulcer histories. Despite this, it is important to note that the difference between the two subgroups was not significant, meaning that amputation and foot ulcer histories were not the major contributors to frail patients’ higher mortality rates.

In terms of severity, the difference between the frail and non-frail groups was significant among participants with mild severity, with frail participants being three times likelier to experience mortality than non-frail participants. Previous studies (58–60) have stated that the severity of the participant cohort might be a more essential contributor to mortality than frailty alone. Interestingly, the severe group did not show the same significance, which contradicts the previous conclusion. However, we only included one study in the severe subgroup, with the aim to obtain a more robust result. Future studies should focus more on patients with more severe conditions.

A similar condition also applied to polypharmacy. As polymedication is often associated with the existence of multiple comorbidities that require pharmaceutical treatments, such as hypertension, COPD, diabetes, CKD, CVD, and cognitive impairment, frailty is not a significant risk factor for mortality under a polypharmaceutical regime. This is supported by the non-significance in the difference between the frail and non-frail groups under a polypharmaceutical regime and the significant difference between the frail and non-frail groups without a polypharmaceutical regime in this meta-analysis result. However, it is still important to note that only one study with a polypharmaceutical regime was included in this meta-analysis.

Finally, in terms of wound types, frail participants with both open wounds and other types of wounds were three times likelier to experience mortality than non-frail participants. This indicates that the association between frail status and mortality was linked with open and other types of wounds. Chronic open wounds were a risk factor for surgical site infection after vascular surgery intervention because of the likelihood of colonized bacteria at the surgical site (84). Thus, wound infection had a serious impact on postoperative mortality and hospital LOS (85, 86).

Overall, the results of the subgroup analysis align with previous findings. Frail patients are even more likely to experience worsened adverse health outcomes if they have older age, undergone major vascular surgeries and experiencing open wounds. To obtain more robust evidence, further studies should focus on polymedication and the connection between frailty and mortality rates in vascular patients.

### Length of hospital stay, discharge location, and rehospitalization

Hospital LOS can be affected by differences in discharge policies and the nature of the patient population (56). A shorter LOS might contribute to a lower in-hospital mortality rate, although patients might perform worse after discharge from surgical units (71). The results of this meta-analysis showed that frail participants were significantly likelier to have longer LOS compared to non-frail participants after vascular surgery, including amputations. However, as indicated by the effect size of 0.217, this association was not strong. It is likely that frail participants need longer to recover from surgical complications after vascular surgery compared to non-frail participants due to their lower ability to cope with physiological changes and to independent living (9, 13). This result aligns with previous studies (61–63), but the requirements of further health care after discharge were rarely considered in previous studies. The subgroup analysis also indicated that patient severity had a significant impact on the association between frailty and LOS.

The results of LOS should be considered together with the discharge location of participants. Whether participants were discharged to their home or other health care facilities, such as rehab units, age care, or even a palliative care unit, can represent a level of independence and vulnerability after vascular surgeries (38, 43, 46). This shows that, although frail participants were likelier to stay in the hospital and receive hospital care for a longer period, they were still more vulnerable and less likely to live independently after discharge. It also suggests that an adverse postoperative health outcome was likelier to affect frail participants for a longer period compared to non-frail participants and that this impact would lead to rehospitalization after index discharge.

This result showed a significantly higher rate of rehospitalization in frail participants. Frail participants were 1.4 times likelier to be hospitalized after index discharge than non-frail participants. The heterogenicity test provided a low *I*^2^ value of 7.993%, indicating that the nine studies (29, 30, 33, 34, 36, 38, 46, 48, 50) reporting rehospitalization had the lowest heterogenicity among all primary outcomes (Table 6). This further supports the hypothesis that frail vascular patients were likelier to be affected by adverse postoperative outcomes for a longer period and required further health care services compared to non-frail patients.

The subgroup analyses for LOS in hospital and rehospitalization also indicate that, with older age, undergoing vascular surgery and having an open wound, frail patients are likelier to experience longer hospital stays and be discharged to a non-home location or be readmitted to the hospital. This is because all the above mentioned contributors are related to a reduction in ability in wound healing and overcoming postoperative complications due to age related reduction in physiological functions and wound related difficulties in recovery (75, 76, 78). In addition, the ability to recover was further worsened by frailty status, as frail patients need a longer time to recover after surgery, which leads to additional requirements of health care services. As the small *I*^2^ indicates, data included in the subgroup analysis of rehospitalization had high heterogenicity (Table 6).

As different vascular surgery patients and amputation patients would response differently to wound healing and postoperative recovery. They would also response differently to adverse health outcomes, and thus the length of hospital stays and rehospitalisation. The subgroup analysis result of both the LOS and rehospitalisation rate showed that comparing to frail amputation patients, frail vascular surgery patients are likelier to stay in hospital longer and being hospitalized. The high heterogenicity within the vascular surgery articles in both LOS and rehospitalisation did bring alter on data reliability, meaning that further analysis is worth doing if articles can be further categories into more specific surgeries. Unfortunately, as the current articles have included multiple different surgeries and articles focused on specific types of surgery are very limited in numbers, no further analysis can be achieved at this point. Despite this, the significant p-values for vascular surgery in both LOS subgroup analysis and rehospitalisation rate subgroup analysis are sufficient to indicate that there are significant stronger links between LOS, rehospitalisation, and frailty among vascular surgery groups.

In conclusion, in addition to preoperative assessment, treatment, recovery, and rehabilitation after vascular surgery are also important domains to consider for minimizing adverse outcomes related to vascular surgery and for improving patient’s postoperative quality of life.

### Secondary outcomes

Comorbidities and chronic conditions are deemed risk factors for adverse health outcomes (12, 87) and are associated with the level of frailty, with some frailty assessment tools considering the incidence of comorbidities as one of the domains contributing to frailty (87). The results of this meta-analysis showed that a vascular surgery patient assessed as frail through frailty assessment tools is significantly likelier to have at least one of the following conditions: postoperative myocardial infarction, postoperative renal failure, CVA and stroke, hypertension, CVD, PVD, diabetes, COPD, or cognitive impairment. This result further indicated the association between frailty and investigated comorbidities in vascular surgery patients, including those who have undergone amputations. It can be concluded that the abovementioned comorbidities are risk factors for frailty and adverse postoperative health outcomes. The result indicates that vascular surgery patients with any of the abovementioned comorbidities are likelier to be frail.

Although most comorbidities were shown to be significantly associated with frailty, CKD is one exception. The results showed that CKD is not significantly associated with frailty. This can be due to the fact that CKD is only defined when the eGFR level is less than 60 mL/min per 1.73 m^2^, while reduced kidney function can occur with a higher eGFR level (88, 89). In addition, studies that included CKD (25, 26, 30, 33, 38, 39, 43, 44) incidence had the highest heterogenicity among all reported outcomes (*I*^2^ = 99.958, *p* < 0.001) (Table 2). As frailty is associated with reduced physiological reserves, sarcopenia is part of the frailty phenotype proposed by Fried et al. (8). With the continuous decline in kidney function and increasing loss of electrolytes, including magnesium, sarcopenia is common in patients with CKD and becomes more severe with the progression of the disease. Thus, frailty is thought to be positively associated with CKD, and patients are expected to be frailer with the progression of the disease (90–92). The contradiction shown in this meta-analysis result can be due to the high heterogeneity among reported CKD outcomes. To explore this association further, future studies should focus on a larger cohort size with lower heterogenicity.

One notable outcome is cognitive impairment. Cognitive impairments, including postoperative cognitive dysfunction and delirium, are associated with deterioration of cognitive performance that presents as reduced memory and concentration (93) and are often associated with chronic pain (94). Delirium is associated with postoperative outcomes. It is an acute and fluctuating disorder characterized by disturbances in concentration, awareness, and cognition (95, 96). It is commonly presented in clinical patients and can develop within a short period of time (96). The inclusion of cognitive impairments and delirium is lacking in previous meta-analyses and reviews (61–63). A previous cohort study focusing on vascular surgery patients (97) concluded that the use of GFI in preoperative evaluation has a positive predictive value for postoperative delirium. However, the study results were limited by the lower-than-expected incidence of postoperative delirium. Thus, in that study’s later multivariate analysis, no significant outcomes were found for GFI. Our meta-analysis included five studies reporting cognitive impairment. The results showed that vascular patients assessed as frail are significantly likelier to experience cognitive impairment both at baseline and after surgery, further indicating the positive predictive values of various frailty assessment tools for postoperative delirium and cognitive impairment. This significant value can be due to the fact that most of the frailty assessment tools have covered the assessment of risk factors of delirium, including comorbidities, activity dependence, weakness, and low energy, thus leading to a positive association between frailty and postoperative delirium.

One of our focused comorbidities was diabetes. It is important to note, however, that in this meta-analysis, studies focusing merely on diabetic patients who underwent amputations were included. This largely included the number of diabetic patients in both the frail and non-frail groups. As diabetic foot ulcers and vascular diseases are often developed along with the progression of diabetes (4, 98), diabetic patients are an important cohort of vascular surgery patients. In this meta-analysis, the results showed that frail patients were significantly likelier to have diabetes at baseline. This might contribute to a subsequently higher risk of developing adverse postoperative health outcomes, such as a higher mortality rate, longer LOS, higher rehospitalization rate, and higher rate of non-home discharge. While this meta-analysis is limited by the number of studies available and the types of exposure reported in the studies, an analysis focusing on diabetic vascular patients can be conducted in the future when more trials or observational studies focusing on diabetic vascular patients are conducted. This would allow us to have a deeper understanding of the association and causal relationship between diabetes, frailty score, and adverse postoperative health outcomes.

### Limitations

As mentioned in previous meta-analyses (61), frailty scores such as mFI and RAI (included in this review) focus on measuring the distinct risk of patients developing adverse health outcomes after operations instead of frailty itself. While frailty and surgical risk are not equivalent concepts, the usefulness of such tools for identifying frailty, and therefore the vulnerability and ability of independent living of patients, is uncertain. While such validated surgical frailty tools would provide more robust assessment results regarding the likelihood of individuals experiencing adverse postoperative health outcomes, they are not direct measurements of the frailty or vulnerability of individuals. Thus, the significant difference seen in the results for which frail patients are likelier to experience adverse health outcomes, such as higher mortality rates, longer hospital stays, higher readmission rates, and higher rates of non-home discharge, might be the result of the internal association between surgery-validated frailty assessment tools and adverse postoperative health outcomes.

For frailty identification alone, both independent frailty scores and risk frailty scores are likely to identify the same patient as frailty as both tools were based on the Fried phenotypes with risk frailty score considered extra adverse health outcome risk factors. With this, if considering the possibility of a certain patient having adverse postoperative outcomes while making clinical decisions on surgery eligibility, both kinds of tools can be used comparably. However, risk frailty scores are more likely to identify someone as frail while independent frailty scores don’t as the presence of risk factors instead of frailty phenotypes might be considered as “frail” in the former but “non-frail” in the latter. Therefore, frailty assessment tools such as the CFS, EFS, FRAIL scale, and Rockwood Frailty Index that are validated for the general population would be referred to and are more reliable for use in both a surgical setting and general practice if the aim is to identify frailty by itself. The use of these tools only accounts for 43% of included studies.

In addition to the use of frailty tools that limit the reliability and accuracy of the results, there are a few other limitations that need to be noted. This meta-analysis included 24 observational studies focusing on frailty as exposure, and no randomized controlled trial (RCT) studies were included. The lack of RCT studies in this area might be due to difficulties in achieving ethical appropriateness, as outcomes are more acute and severe for cases that need urgent surgical treatments. All the included studies were medium to high quality according to the JBI Checklist for Cohort Studies and yielded good-quality results with minimal publication bias. Nevertheless, one significant limitation of this study was the high heterogenicity of the included studies. This is likely to be due to the difference in the types of surgery studied, the number of participants included, the length of follow-up, and the heterogeneous population profile. The high heterogenicity in data can be contributed to the wide range of surgeries study participants have taken. As mentioned in the method section, vascular surgery covers a wide range of different surgery, both open and endovascular surgery. These surgeries can affect patients differently but given most of the selected studies have reported mixed results, subgroup analysis on each surgery type separately was difficult to achieve. Future meta-analyses involving subgrouping and analyzing different vascular surgery in detail can be considered if more single surgery focused primary studies have emerged. Another limitation was that the study populations were predominantly male (64% of the total population), which may have caused bias relating to the representation of the study population. As previously evidenced in various studies, being male is one of the risk factors for experiencing comorbidities including hypertension, diabetes, and CVD (99–103). Thus, being male dominated could bias the association between comorbidities and frailty. Given these limitations, the results of this study should be interpreted with caution.

### Strengths

This meta-analysis has explored multiple databases to include as many relevant studies as possible and is the first meta-analysis to include the most recent studies published between 2020 and 2022. After the first-round reviews, an ongoing review of article reference lists and newly published articles was conducted to include all eligible articles up to date. The final analysis included 24 medium- to high-quality studies, which contributed to a robust and reliable result.

Furthermore, detailed and comprehensive subgroup analyses were included in this study. Multiple health outcomes were included in these studies, and many of the comorbidities investigated, including postoperative myocardial infarction, renal failure, stroke and CVA, COPD, cognitive functions, and delirium, and eGFR have not been included or systematically reviewed in previous studies. By reviewing these outcomes, this study has provided a more comprehensive overview of the associations between frailty defined by different frailty assessment tools and postoperative health outcomes that link frailty to comorbidities associated with multiple different organs and physiological systems.

In addition, with careful and very specific considerations of the eligibility criteria, all studies included used validated frailty tools and grouped participants based on their frailty status. The significant result from the analysis of these studies is more robust and further strengthens the idea that frailty is significantly associated with adverse health outcomes, with validated frailty assessment tools being able to identify frailty status. It predicts possible adverse postoperative outcomes, including mortality, longer hospital stays, more unplanned rehospitalization, and higher non-home discharge.

## Conclusion

This meta-analysis suggests that frailty status identified by validated frailty assessment tools is significantly associated with adverse postoperative health outcomes and the development of comorbidities in vascular surgery patients who underwent various types of vascular surgeries, including amputations. Frail vascular surgery patients are likelier to experience higher in-hospital and postdischarge mortality, longer hospital LOS, higher rates of non-home discharge that require further health care services, and higher rates of rehospitalization. This could be attributed to the significant associations between frailty and comorbidities, including postoperative myocardial infarction, postoperative renal failure, CVA and stroke, hypertension, CVD, PVD, diabetes, COPD, cognitive impairment, and lower eGFR levels. However, surgery-validated frailty scores, such as mFI and RAI, should be used with caution when investigating the association between frailty and adverse health outcomes, as these tools are highly related to the risk of developing adverse health outcomes after surgery. Frailty scores such as CFS, EFS, Rockwood Frailty Index, and the FRAIL scale are more reliable for use in research in both a surgical setting and in general practice.

## Data availability statement

The original contributions presented in this study are included in this article/supplementary material, further inquiries can be directed to the corresponding author.

## Author contributions

MJ and NM contributed to the conceptualization of the study, the study design, and the revision of the manuscript. SC contributed to the study design, literature search, quality assessment, and data extraction and wrote the manuscript. RD contributed to the literature search and quality assessment. JS contributed to the study design, data analysis, and supervision of the research team and wrote, edited, and reviewed the manuscript. All authors contributed to the article and approved the submitted version.
